# Neuraminidase Inhibition Primes Short-Term Depression and Suppresses Long-Term Potentiation of Synaptic Transmission in the Rat Hippocampus

**DOI:** 10.1155/2015/908190

**Published:** 2015-01-31

**Authors:** Alina Savotchenko, Arthur Romanov, Dmytro Isaev, Oleksandr Maximyuk, Vadym Sydorenko, Gregory L. Holmes, Elena Isaeva

**Affiliations:** ^1^Department of Cellular Membranology, Bogomoletz Institute of Physiology, Bogomoletz Street 4, Kiev 01024, Ukraine; ^2^Department of Neurological Sciences, University of Vermont, 149 Beaumont Avenue, Burlington, VT 05405, USA

## Abstract

Neuraminidase (NEU) is a key enzyme that cleaves negatively charged sialic acid residues from membrane proteins and lipids. Clinical and basic science studies have shown that an imbalance in NEU metabolism or changes in NEU activity due to various pathological conditions parallel with behavior and cognitive impairment. It has been suggested that the decreases of NEU activity could cause serious neurological consequences. However, there is a lack of direct evidences that modulation of endogenous NEU activity can impair neuronal function. Using combined rat entorhinal cortex/hippocampal slices and a specific inhibitor of NEU, 2-deoxy-2,3-dehydro-N-acetylneuraminic acid (NADNA), we examined the effect of downregulation of NEU activity on different forms of synaptic plasticity in the hippocampal CA3-to-CA1 network. We show that NEU inhibition results in a significant decrease in long-term potentiation (LTP) and an increase in short-term depression. Synaptic depotentiation restores LTP in NADNA-pretreated slices to the control level. These data suggest that short-term NEU inhibition produces the LTP-like effect on neuronal network, which results in damping of further LTP induction. Our findings demonstrate that downregulation of NEU activity could have a major impact on synaptic plasticity and provide a new insight into the cellular mechanism underlying behavioral and cognitive impairment associated with abnormal metabolism of NEU.

## 1. Introduction

Long chains of negatively charged sialic acid occupy a prominent position on cellular membrane proteins in complex carbohydrates, which are major constituents of membrane proteins and lipids and are involved in manifold cell signaling events [1]. In the central nervous system, sialic acids play an important role in many processes such as neurogenesis, cell differentiation, migration, axon sprouting, synaptogenesis, plasticity, and neuronal excitability [2, 3]. Involvement of polysialic acid (PSA), a homopolymer of sialic acid, in a wide range of neuronal functions, related to the ability of PSA to modulate attracting and repulsing molecule-molecule interactions and membrane surface charge density due to their negative charge, bulky size, and location on the outer surface of the membrane [4, 5].

The physiological role of sialic acid comes from studies using neuraminidase (NEU) as an enzyme, which hydrolyzes terminal sialic acid residues from cellular glycoconjugates. In most studies, NEU is applied extracellularly to decrease cell sialylation [2, 6–9]. Removal of sialic acid by NEU affects neurogenesis, synaptogenesis, synaptic plasticity, neuronal excitation, and spatial learning and causes behavioral abnormalities [2, 6, 10–12]. Changes of endogenous NEU activity, as a physiological regulator of the level of sialic acid, could also alter neuronal function. Clinical observations indicate that an imbalance in the metabolism of NEU has a significant influence on the function of neuronal systems. Indeed, mental retardation and seizures are common clinical features of inherited disorders of defective or deficient NEU activity [13, 14]. Various pathological conditions such as chronic stress, seizure activity, and chronic ethanol treatment induce changes in NEU activity in different regions of the brain [15–17]. These changes in NEU activity have been suggested to be responsible for physiological and neurological impairment in the brain, presumably due to the effect of NEU on glycosylation [18]. However, there is a lack of direct experimental studies showing that alteration of endogenous NEU activity could affect neuronal function.

Previous* in vitro *and* in vivo *studies show that decreases of NEU activity by ganglioside sialidase inhibitors induce synaptogenesis and affect neuronal excitation, which is accompanied by the accumulation of PSA in rat hippocampus [2, 19, 20]. As the deficit of NEU activity is associated with behavior and mental impairment and alterations of the level of PSA and neuronal network activity alter synaptic plasticity, we examined the consequences of NEU inhibition on synaptic plasticity as a cellular basis for behavioral and cognitive functions. Using the specific ganglioside sialidase inhibitor 2-deoxy-2,3-dehydro-N-acetylneuraminic acid (NADNA), we show that short-term blockade of endogenous NEU alters long-term synaptic potentiation (LTP) and frequency dependent plasticity in combined entorhinal cortex/hippocampal slices without an effect on paired-pulse facilitation and long-term depression (LTD). Application of low-frequency stimulation before the induction of LTP restores LTP in NADNA-pretreated slices to the control level. This data indicates that NADNA treatment induces potentiation of synaptic response, which results in the decrease in the magnitude of subsequent LTP.

## 2. Methods

All experimental procedures were performed in accordance with the guidelines set by the National Institutes of Health for the humane treatment of animals and approved by the Animal Care Committee of Bogomoletz Institute of Physiology.

### 2.1. Slice Preparation

Combined entorhinal cortex/hippocampal slices including neocortical areas (Te2 and Te3), entorhinal cortex, subiculum, and hippocampus were prepared from Wistar rats aged 19 to 21 days postnatally (P19–21) as previously described with some modifications [21]. On the day of the experiment the rat was deeply anesthetized using isoflurane and decapitated. The brain was removed and placed into ice-cold oxygenated (95% O_2_-5% CO_2_) artificial cerebrospinal fluid (ACSF) of the following composition (mM): NaCl 119, KCl 2.5, CaCl_2_ 2.0, MgSO_4_ 1.3, NaHCO_3_ 26, NaH_2_PO_4_ 1.2, and glucose 11 (pH 7.35). Cerebellum, frontal lobe region (coronal section), and ventral-lateral areas (sections at the angle 20° to 30° off the horizontal axis) were removed from the brain. The remaining part of the brain was mounted on the stage of a Vibroslice NVSL (World Precision Instruments Inc., Sarasota, FL, USA) and cut (400 *μ*m) through the hemispheres at an angle of 30–35° of their horizontal planes. For the experiments we took 3-4 slices from the dorsal part of the hippocampus [22]. Slices were maintained in an oxygenated ACSF at a room temperature for at least 1.5 h before use.

### 2.2. Electrophysiology

Brain slices were transferred to the incubation chamber and superfused with oxygenated ACSF at a rate of 2 mL/min (22–24°C). Extracellular recordings were obtained within the CA1 stratum radiatum (SR) of hippocampus with extracellular glass microelectrodes (3-4 MΩ) filled with ACSF using patch-clamp amplifier (PC 501A, Warner Instruments Corp., Hamden, CT). Stimulating and recording electrodes were placed on the slice surface approximately 400 *μ*m apart from each other. Evoked postsynaptic responses were elicited by stimulation of Schaffer collateral-commissural pathway using a concentric bipolar stimulating electrode (FHC Inc., Bowdoin, ME) connected to a flexible stimulus isolator (ISO-Flex, A.M.P. Instruments, Jerusalem, Israel). Stimulation intensity varied between 150 and 400 *μ*A in all slices. At the beginning of each experiment maximal synaptic response was determined by generating input–output curves. For baseline recording the current intensity was set to elicit 30% of maximal response. The stimulation was applied every 30 s. The stimulation protocol to induce synaptic plasticity was applied after 10–20 min of stable baseline recording. In paired-pulse experiments two stimuli were delivered to the hippocampal pathway with interstimulus intervals (ISI) ranging from 25 to 300 ms. The paired-pulse ratio was defined as S2/S1, where S1 and S2 are slopes of the postsynaptic responses evoked by first and second pulses, respectively. To induce LTP either high-frequency tetanic stimulation (HFS) of 100 pulses at frequency of 100 Hz or a brief tetanic stimulation (15 pulses at 50 Hz) was delivered at baseline stimulation intensity. Short-term depression (STD) was measured during 100 Hz and 50 Hz trains. To induce LTD low-frequency stimulation (LFS) of 1800 pulses at frequency of 1 Hz was delivered at baseline stimulation intensity. Recordings were digitized at 10 kHz using an analogue-to-digital converter (National Instruments, Austin, TX) and stored on a computer using the WinWCP program (Strathclyde Electrophysiology Software, University of Strathclyde, Glasgow, UK).

### 2.3. NEU Blocker Treatment

Brain slices were incubated with NADNA during 2 hr at room temperature then extensively washed with ACSF before recordings. In all experiments we used NADNA in concentration of 500 *μ*M purchased from Sigma-Aldrich (St. Louis, MO, USA). The specificity of the effect of NADNA as a blocker of the endogenous NEU was shown in histological and electrophysiological studies in our previous reports [2, 19].

### 2.4. Drugs

D-2-Amino-5-phosphonopentanoic acid (D-APV) was obtained from Tocris (Ellisville, MO, USA). All other chemicals were purchased from Sigma (St. Louis, MO, USA).

### 2.5. Data Analysis

Offline analysis of the recordings was performed using Clampfit (Axon Instruments, USA), Prism 5 (GraphPad, La Jolla, CA), and Origin 7.5 (OriginLab, Northampton, MA) software. Statistical comparison of the effects of NADNA treatment on different forms of plasticity (except STD during 100 Hz train) was performed by measuring the initial slope of the field response. The average field excitatory postsynaptic potential (fEPSP) slope during a 5 min period before LTP and LTD induction was taken as the baseline, and all values were normalized to this baseline. LTP magnitude was measured from 30 to 55 min after tetanic stimulation (during this time fEPSP was typically stable). In experiments where LFS preceded HFS, LTP magnitude was measured from 20 to 30 min after tetanic stimulation. The magnitude of fEPSP depression induced by LFS was estimated from 20 to 30 min after stimulation. During the above mentioned periods normalized fEPSP slopes were averaged for each slice and the mean values were then averaged and compared for control and NADNA-pretreated group. For measurement of STD during 50 Hz train all fEPSP slopes were normalized to fEPSP slope in response to the first pulse. The synaptic response to 100 Hz train stimulation was measured as a single entity and normalized to the prestimulus baseline fEPSP amplitude for each recording. The magnitude of STD was estimated during the last 100 ms of the train and averaged for each slice. Obtained mean values were then averaged for control and NADNA-pretreated groups and compared. Fiber volley (FV) amplitude was measured as a difference between the positive and the following negative peak. In experiments, where fEPSPs were blocked with glutamate receptor antagonist before HFS, FV amplitudes were normalized to the prestimulus baseline FV amplitude for each recording. Two-way repeated measures ANOVA and unpaired Student's* t*-test were used to analyze difference between groups. A *P* value less than 0.05 was considered significant. Results were expressed as Mean ± SEM; *n* is the number of slices.

## 3. Results

**Figure 1:**
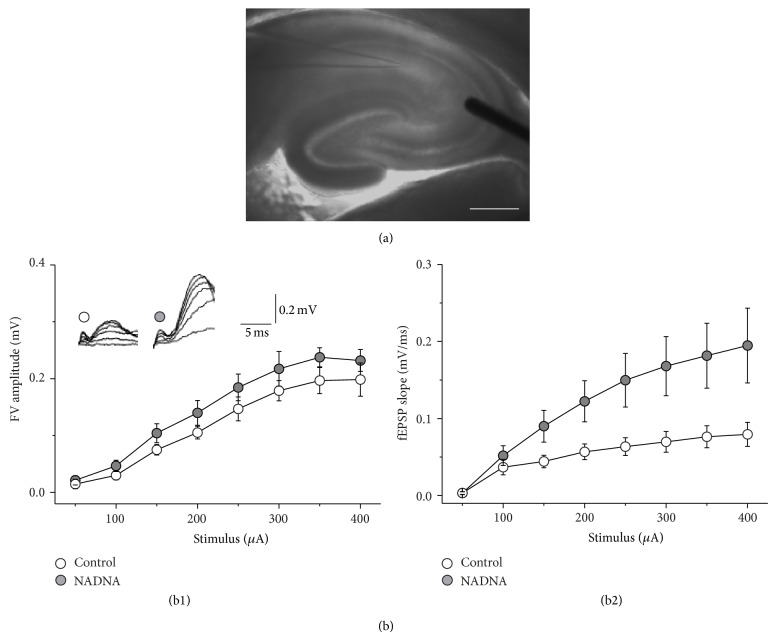
Inhibition of NEU activity affects basal evoked excitatory synaptic transmission. (a) The arrangement of electrodes for recordings. fEPSPs were evoked by stimulation of Shaffer collaterals using concentric electrode (right) and recorded using a glass electrode (left) filled with extracellular solution placed in CA1 SR region. Scale bar, 0.5 mm. (b) Input-output curves show significant increase of the maximal fEPSP slope after suppression of NEU activity without the effect on the fiber volley (FV) amplitude.

**Figure 2:**
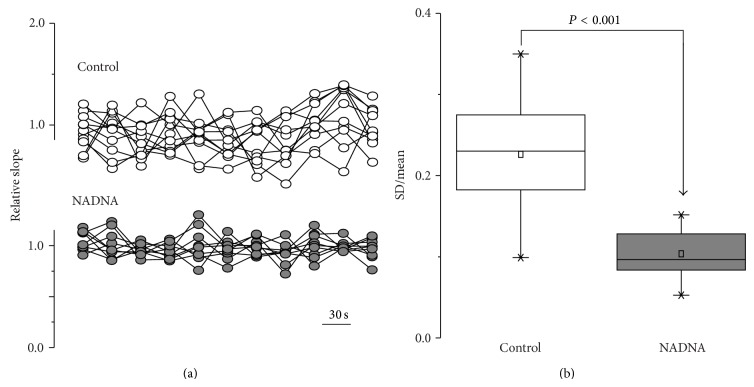
Effect of inhibition of NEU activity on variation of field potential response in hippocampal CA3-to-CA1 network. (a) Variation of baseline fEPSP recorded at 0.033 Hz in control and NADNA-pretreated slices. Each curve represents normalized to average baseline fEPSP slope from one slice. (b) Histogram shows that variation of baseline fEPSP slope significantly decreases in slices pretreated with NADNA.

Previously we showed that blockade of NEU activity leads to an increase in the density of simple and perforated synapses in hippocampal CA1 SR region [19]. To test whether newly formed synapses are functional, Shaffer collaterals were stimulated and field potential recordings were performed from the CA1 SR region in control and NADNA-pretreated slices (Figure 1(a)). To estimate the maximal field potential response in each recording the stimulation intensity was gradually increased until the amplitude of the response reached the saturation level. Input/output curves revealed a significant increase of the maximal rising slope of fEPSP in NADNA-pretreated slices compared to controls (NADNA-pretreated group: 0.20 ± 0.05 mV/ms [*n* = 21]; control: 0.08 ± 0.02 mV/ms [*n* = 17], *t*_36_ = 2.1, *P* < 0.05, Figure 1(b)(b2)) without alteration of FV amplitude (NADNA-pretreated group: 0.22 ± 0.01 mV [*n* = 11]; control: 0.20 ± 0.01 mV [*n* = 17],* t*_19_ = 0.9, *P* = 0.34, Figure 1(b)(b1)). The coefficient of variation of the baseline fEPSP slope (30% of the* maximal* response) was significantly decreased in the NADNA-pretreated group compared to controls: SD/Mean, 0.22 ± 0.02 [*n* = 11] in control versus 0.10 ± 0.04 [*n* = 9] after pretreatment with NADNA (*t*_18_ = 4.83, *P* < 0.001, Figure 2). FEPSPs consist of N-methyl-D-aspartate (NMDA) and non-NMDA receptor-mediated components. To clarify whether NADNA pretreatment differently affects these components, we used specific NMDA receptor antagonist D-2-amino-5-phosphonopentanoic acid (D-APV) and non-NMDA receptor antagonist 6-cyano-7-nitroquinoxaline-2,3-dione (CNQX). Application of 50 *μ*M D-APV produced a similar modest decrease in the baseline synaptic response in both groups (91.1 ± 3.5% [*n* = 9] in control versus 85.8 ± 5.0% [*n* = 8] in NADNA-pretreated slices). The remaining component of fEPSP was completely attenuated by application of 10 *μ*M CNQX. These data suggest that NADNA pretreatment similarly affects NMDA and non-NMDA components of synaptic responses.

**Figure 3:**
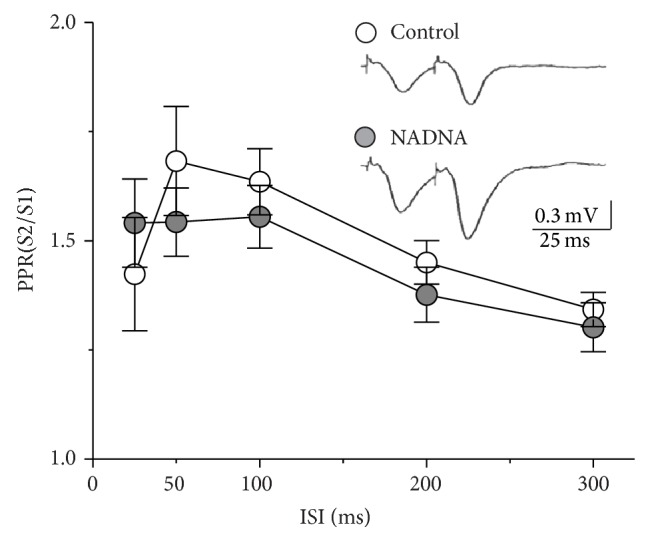
NEU inhibition does not influence paired-pulse plasticity in CA1 region of hippocampus. Histogram of average paired-pulse ratios as a function of ISI in control (white) and NADNA-pretreated (grey) slices. There were no significant differences between groups. Insets: averaged sample records of fEPSP at CA3–CA1 synapses measured at 25 ms ISI. All data are presented as Mean ± SE.

In the next set of experiments we investigated the effect of downregulation of NEU activity on the paired-pulse plasticity as a form of short-term synaptic plasticity reflecting changes in the release probability of presynaptic cell. Depending on the presynaptic release probability, repetitive stimulation could elicit either facilitation or depression [23]. Paired-pulse facilitation (PPF) of synaptic responses in NADNA-pretreated group was recorded in all slices irrespective of the duration of ISI. In control slices, paired-pulse depression of fEPSP was observed at ISI 25 and 50 ms in only two recordings out of twelve (16.7%). At ISI 100 to 300 ms PPF of synaptic responses was observed in all control slices.  Figure 3 indicates that regardless of the ISI the paired-pulse ratio (PPR) of postsynaptic responses did not change after NADNA pretreatment (control group *n* = 12; NADNA-pretreated group *n* = 10; *F*_1,80_ = 0.35, *P* = 0.56).

**Figure 4:**
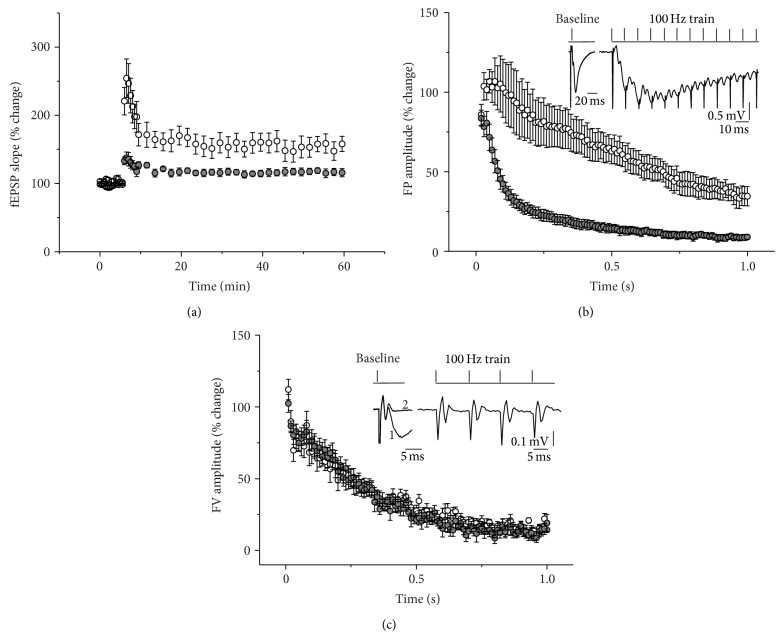
Effect of inhibition of NEU activity on the long-term potentiation and the short-term depression in hippocampal CA3-to-CA1 network induced by 100 Hz stimulus train. (a) Average of baseline-normalized initial slopes of fEPSP evoked by stimulation of Shaffer collateral before and after delivery of 100 Hz train in control (white) and NADNA-pretreated slices (grey). (b) Summarized changes in field potentials recorded during 100 Hz stimulation in control (white) and NADNA-pretreated slices (grey). Each point in the graph represents averaged amplitude of the field potential recorded at the time point of 9.5 ms after every stimulus delivery during train and normalized to baseline fEPSP amplitude. Inset: examples of baseline field potentials and fragment of field potential recording during 100 Hz stimulation from the same slice. (c) Summarized changes of fiber volley (FV) amplitude during 100 Hz stimulation in control (white) and NADNA-pretreated slices (grey) recorded in the presence of NMDA and non-NMDA receptor antagonists (resp., D-APV and CNQX). Each point in the graph represents averaged FV amplitude recorded after every stimulus delivery during the train and normalized to baseline FV amplitude. Inset: examples of baseline field potentials recorded from NADNA-pretreated slice before (1) and after (2) application of glutamate receptor blockers and fragment of field potential recording during 100 Hz stimulation from the same slice. All data are presented as Mean ± SE.

To study the effect of NEU inhibition on long-term synaptic plasticity we used a protocol for LTP induction consisting of 100 stimuli delivered at 100 Hz.  Figure 4(a) represents the group data of fEPSP evoked by stimulation of Shaffer collateral and recorded in CA1 SR region in the control and NADNA-pretreated groups before and after delivery of the HFS. An average maximal posttetanic potentiation was significantly decreased in the NADNA-pretreated group compared to controls (254.2 ± 28.3% [*n* = 10] of baseline in the control group and 137.8 ± 8.4% [*n* = 9] of baseline in the NADNA-pretreated group, *t*_17_ = 3.76, *P* = 0.002). There was also a significant difference in LTP magnitude between NADNA-pretreated (115.6 ± 4.9% [*n* = 9]) and control (155.0 ± 13.2% [*n* = 10]) groups (*F*_1,850_ = 7.18, *P* = 0.02).

During the high-frequency stimulus train fEPSP exhibited a strong depression.  Figure 4(b) illustrates group data of the dynamic of evoked field potential changes during delivery of 100 pulses at 100 Hz in both groups. The relative field potential amplitudes at the end of the train (estimated during the last 100 ms of the train) were 37.2 ± 0.4% [*n* = 10] in control versus 8.8 ± 0.1% [*n* = 9] in NADNA-pretreated slices (*F*_1,153_ = 25.6, *P* < 0.0001).  Figure 4(c) shows that no difference in FV amplitude dynamic between control and NADNA-pretreated groups during HFS was observed in experiments where NMDA and non-NMDA receptor antagonists (50 *μ*M D-APV and 10 *μ*M CNQX) were applied before HFS. These findings indicate that changes of STD level in the NADNA-pretreated group are due to the alteration of synaptic machinery.

**Figure 5:**
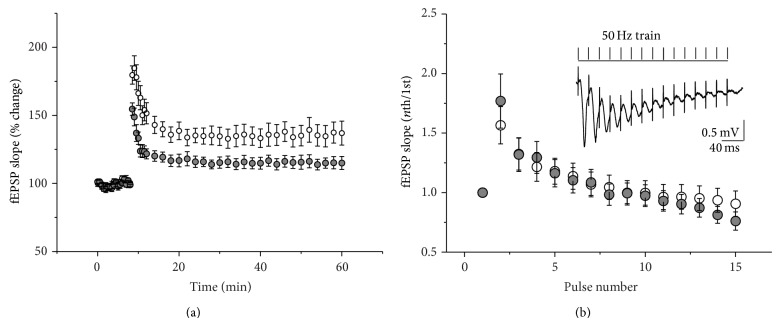
Effect of inhibition of NEU activity on the long-term potentiation and the short-term depression in hippocampal CA3-to-CA1 network induced by 50 Hz stimulus train. (a) Average of baseline-normalized initial slopes of fEPSP evoked by stimulation of Shaffer collateral before and after delivery of 50 Hz train in control (white) and NADNA-pretreated slices (grey). (b) Cumulative changes in field potentials recorded during 50 Hz stimulation in control (white) and NADNA-pretreated slices (grey). fEPSP slopes were normalized to the fEPSP slope in response to the first pulse and graphed versus pulse number. Inset: field potential recording during 50 Hz HFS.

To eliminate possible effects of depression during prolonged HFS on the posttetanic response, in the next set of experiments we used a brief tetanic stimulation protocol (15 pulses delivered at 50 Hz) to induce synaptic potentiation [24]. In both groups repetitive stimuli evoked an initial facilitation of the fEPSP response followed by their gradual decrease with time. There was no significant difference in STD between groups (Figure 5(b), *F*_1,504_ = 0.02, *P* = 0.88). However, there was a significant difference in the maximal posttetanic potentiation between groups (controls: 179.7 ± 6.7% [*n* = 17] of baseline; NADNA-pretreated group: 154.6 ± 4.5% [*n* = 21] of baseline, *t*_36_ = 3.2, *P* = 0.003). Also, we found significant difference in the magnitude of LTP in the NADNA-pretreated group (115.2 ± 4.2% [*n* = 21]) compared to controls (136.5 ± 7.7% [*n* = 17], *F*_1,864_ = 6.58, *P* = 0.01, Figure 5(a)).

**Figure 6:**
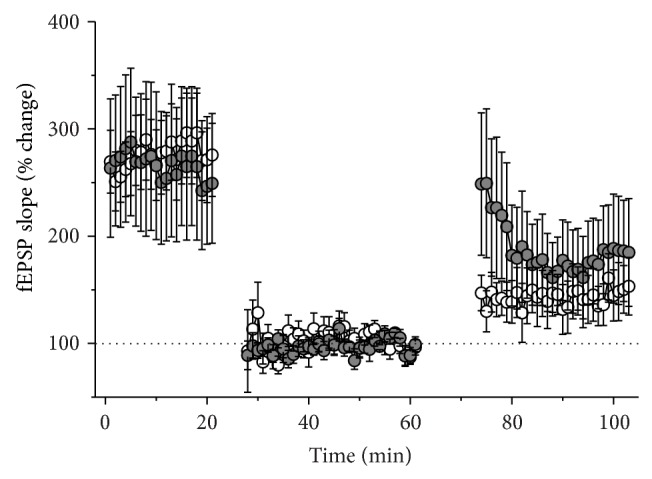
Synaptic depotentiation restores LTP in NADNA-pretreated slices to the control level. Pretreatment of slices with NADNA did not affect LTD of fEPSP evoked by LFS stimulation. Thirty min after the induction of LTD, tetanic stimulation of the Shaffer collaterals induces a similar potentiation of the field potential responses in NADNA-pretreated and control slices. All data are presented as Mean ± SE.

Previous reports show that the downregulation of NEU activity and the increase in tissue sialylation enhance neuronal activity [2, 19, 20, 25, 26]. The increase in neuronal activity was shown to produce LTP-like changes [27], which can lead to the impairment of subsequent LTP induction. To examine the possibility that the decrease in LTP magnitude in NADNA-pretreated slices can be due to the saturation of LTP produced by increased neuronal activity we delivered LFS (1800 pulses at 1 Hz) before applying the LTP protocol (100 pulses at 100 Hz). LFS induces a similar reduction of fEPSP slope in both groups (LTD magnitude for control: 48.0 ± 6.8% [*n* = 19] of baseline and for NADNA-pretreated group: 58.0 ± 12.9% [*n* = 9] of baseline, *F*_1,234_ = 0.57, *P* = 0.5). After obtaining a new stable baseline (baseline 2; 20–30 min after LFS) high-frequency tetanic stimulation was applied. HFS induces the potentiation of postsynaptic responses in both groups. In these experiments we did not find statistically significant group differences in the LTP magnitude (control: 145.6 ± 22.1% [*n* = 19] of baseline 2 and NADNA-pretreated group 180.6 ± 43.7% [*n* = 9] of baseline 2, *F*_1,234_ = 0.64, *P* = 0.4, Figure 6).

## 4. Discussion

The present study demonstrates that suppression of endogenous NEU activity with the specific inhibitor NADNA (i) causes an increase in the field potential slope and a reduction in variation of fEPSP in CA3-to-CA1 network of rat hippocampus, (ii) does not alter paired-pulse plasticity and LTD, (iii) promotes STD during prolonged HFS, and (iv) decreases LTP magnitude; and (v) synaptic depotentiation restores the LTP deficit in NADNA-pretreated slices to the control levels. These data show that changes in the activity of endogenous NEU can substantially modify hippocampal synaptic plasticity which may be the cellular correlate of behavioral and cognitive impairment associated with abnormal metabolism of NEU and altered PSA levels [13, 28, 29].

Significant increases in the proportion of perforated synapses in the CA1 SR region of hippocampus have been observed in cultured hippocampal slices pretreated with NADNA [19]. Alterations in the density of perforated synapses are considered as the morphological feature of activity-dependent synaptogenesis and have been reported under different physiological and pathological conditions [30–32]. In the present study we observed that NEU inhibition significantly increases postsynaptic responses while decreasing their variation. Such an effect is observed under condition of changes in neuronal excitability and following LTP induction [33]. Our data demonstrates that morphological changes caused by suppression of endogenous NEU activity parallel the enhanced functional excitatory synaptic connectivity in the hippocampal CA3-to-CA1 network. Additionally, direct PSA-AMPA receptor interactions were recently reported using exogenously applied bacterial PSA, colominic acid [34]. As AMPA receptor activity is the main contributor of fEPSP, we cannot exclude the possibility that increases in fEPSP may be partly due to direct potentiation of AMPA receptors through the increase in sialylation level caused by downregulation of NEU activity.

Inhibition of NEU activity did not affect paired-pulse plasticity. This data is in agreement with previous study where modulation of the PSA level by exogenously applied NEU did not affect short-term plasticity in CA3-to-CA1 networks of organotypic hippocampal culture [9]. In addition, pretreatment with NADNA did not affect LTD. Similar results were obtained previously using another NEU blocker, oseltamivir carboxylate, the active form of oseltamivir [35]. However, NADNA-pretreated slices show more pronounced depression in response to continuous HFS than control slices. Prolonged high-frequency stimulation leads to the transient decrease in synaptic strength due to vesicle depletion, inactivation of both release sites and calcium channels [36]. Another possible mechanism of depression during HFS is desensitization of postsynaptic receptors and activity-dependent receptor internalization [37, 38]. As NADNA pretreatment results in synaptic potentiation, both presynaptic and postsynaptic mechanism of STD during HFS could be altered in NADNA-pretreated slices.

In our study significant decrease in LTP magnitude was observed suggesting downregulation of synaptic effectiveness in conditions of NEU inhibition. Previous studies indicate that increase in membrane sialylation or downregulation of NEU activity could significantly alter neuronal activity. Increase of the sialylation level has been proposed to contribute to enhanced neuronal excitability after nerve injury [25]. Inhibition of endogenous NEU was reported to enhance neuronal synchronization in rat hippocampal CA3 region, increase the firing frequency and amplitude of spontaneous synchronous oscillations observed in CA1 region of cultured hippocampal slices, and intensify seizure-like activity in different* in vitro* and* in vivo *models of seizures [2, 19, 20, 26]. It is well recognized that the ability of synaptic pathways to respond to stimulation with LTP is greatly dependent on the background activity as well as previous activation [39]. LTP can be more easily induced in conditions of decreased activity, while induction of LTP will be more difficult in synaptic pathways with a history of increased activity. Indeed, induction of LTP can be inhibited if weak stimulation is previously delivered to the same input pathway [40] or in Mg^2+^-free extracellular solution [41]. Also, learning is accompanied by reduced capability to induce LTP [42–44]. We hypothesize that NEU inhibition through modification of the neuronal activity produces LTP-like changes in the CA1 region of hippocampus, which results in a decrease in magnitude of subsequent LTP. Our experiments, where synaptic depotentiation completely restores LTP deficit in NADNA-pretreated slices to the control level, support this assumption.

Many studies show substantial contributions of PSA in plasticity and memory processing in both the developing and mature brain [29]. The peak of the expression of PSA is correlated with the critical period of neuronal development and PSA has been shown to play an important role in developmental structural and synaptic plasticity. In the adult brain, substantial PSA expression was found in restricted brain regions of continuous neurogenesis including hippocampus, amygdala, and neocortex. These areas have been shown to play an important role in different aspects of memory [10]. Decreases in the level of membrane sialylation impair spatial memory and suppress LTP in hippocampal slices [6, 9, 12]. Impairment of learning and long-term plasticity in CA3–CA1 synapses was demonstrated in mice lacking the polysialyltransferase, an enzyme essential for PSA biosynthesis [10]. On the other hand, exogenous application of PSA impairs LTP and formation of hippocampus-dependent contextual memory [45]. Elevated PSA levels have been associated with various neuropsychiatric disorders such as temporal lobe epilepsy, Alzheimer disease, and chronic stress [46–48]. Short-term inhibition of NEU activity results in an increase of PSA level and neuronal excitability and leads to activity-dependent synaptogenesis in CA1 region of hippocampus [2, 19, 26]. We suggest that impairment of LTP shown in the present study is a consequence of these alterations. These findings indicate that rigorous regulation of the PSA level (regardless of the direction) is a necessary component for normal LTP induction.

## 5. Conclusions

The present study emphasizes the importance of proper functioning of endogenous NEU in neuronal tissue. Our results support the idea that impairment of NEU activity affects hippocampal plasticity and provides insight into the cellular mechanisms underlying inherited disorders of impaired metabolism of NEU and neuropsychiatric disorders followed by changes in membrane sialylation and NEU activity. Moreover, considering that NEU inhibitor derivatives are widely used as anti-influenza drugs and have been reported to cause serious psychiatric side effects in patients receiving these drugs, our data can be used to better understand a possible mechanism of adverse effects of these antiviral drugs and hopefully improve their quality [28, 49].
